# On the Orbital Angular Momentum Incident Fields in Linearized Microwave Imaging

**DOI:** 10.3390/s20071905

**Published:** 2020-03-30

**Authors:** Santi Concetto Pavone, Gino Sorbello, Loreto Di Donato

**Affiliations:** Department of Electrical, Electronics and Computer Engineering (DIEEI), University of Catania, viale A. Doria 6, 95125 Catania, Italy; santi.pavone@unict.it (S.C.P.); gino.sorbello@unict.it (G.S.)

**Keywords:** linearized inverse scattering, microwave imaging, orbital angular momentum, born approximation, rytov approximation

## Abstract

Orbital angular momentum (OAM) is gaining great attention in the physics and electromagnetic community owing to an intriguing debate concerning its suitability for widening channel capacity in next-generation wireless communications. While such a debate is still a matter of controversy, we exploit OAM generation for microwave imaging within the classical first order linearized models, i.e., Born and Rytov approximation. Physical insights into different fields carrying *ℓ*-order OAM are conveniently exploited to propose possible alternative imaging approaches and paradigms in microwave imaging.

## 1. Introduction

Microwave imaging (MWI) deserves great attention in the electrical engineering community due to its potential applications as disparate as subsurface and planetary exploration (ground penetrating radar), non-destructive testing (NDT), biomedical imaging, and so on and so forth [1,2,3]. However, while huge efforts have been addressed in recent years towards the development of experimental equipment, which jointly exploits the availability for relatively low cost microwave devices and the ever increasing computing power of modern CPUs [4,5], the underlying inverse scattering problem (ISP) solution still requires great efforts from the methodological and modeling point of view. This is related to two main features of ISPs: non-linearity and ill-posedness. Indeed, if both of them are not properly faced, MWI cannot be used in any practical instance. To face them, a priori information can be exploited in many different ways to obtain reliable inversion or optimization strategies equipped with effective regularization schemes [6,7,8].

Generally speaking, microwave imaging approaches can be grouped into two main classes: quantitative and qualitative approaches. The first one aims to retrieve both electromagnetic and geometric features, whereas the second one allows only shape characterization of an unknown scattering system in a surveyed region. In general, quantitative methods require the solution of a non-linear problem that inherently results in non-trivial issues, such as local minima [9,10] and regularization of non-convex problems. On the other hand, qualitative methods make a trade-off between the difficulty of solving a non-linear problem and the limited amount of information to be conveyed back from scattered field data. Obviously, a plethora of hybrid methods coexist in the literature, wherein several stepwise optimization strategies, regularization approaches, and approximate models have been proposed to face the inverse scattering problem. Very recently, also machine learning (ML) and deep learning (DL) have been applied to inverse scattering problems [11,12].

Besides the above, with reference to the present paper, it is worth mentioning a recently proposed paradigm for solving inverse scattering problems. Such a paradigm stems from the design of new scattering experiments to recast the original ones into new “virtual” experiments by means of a suitable “design equation”. This latter entails a simple linear recombination of scattered field data, which, owing to the linearity of scattering experiments, enforces the total electric field to be tailored with a specific distribution within the imaging domain. Since these scattering experiments are designed without additional measurements (i.e., only via software processing), they have been named virtual experiments (VE) [13,14,15,16,17]. All these approaches have shown an enhanced capability in imaging scatterers not belonging to the weak scattering regime, while facing the problem in a simpler, or more effective/efficient way, than many other imaging approaches currently available in the state-of-the-art.

In this view, other papers have investigated the use of different kinds of probing fields in microwave imaging. For example, in [18], orbital angular momentum (OAM) incident fields of order higher than ℓ=0, generated through a planar array, were exploited to perform 3D imaging with an observed improvement in the resolution beyond the Rayleigh limit. In [19], sub-wavelength focused near-field (NF) beams and Bessel beams [20,21,22] were proposed to perform imaging in scenarios when possible undesired scatterers are present, and in [23], OAM incident fields were used for accurate recovery of sparse objects through mask-constrained sparse reconstruction.

In order to investigate the capability of OAM antennas in microwave tomographic imaging, possibly exploiting additional degrees of freedom carried by the topological charges of such fields, in this paper, we consider the use of OAM incident fields generated by properly feeding a cylindrical array of filamentary currents. Specifically, the “view diversity” conventionally exploited in the scattering experiments is traded with the “mode diversity” carried by OAM incident fields of different order. In doing this, we adopt linearized imaging procedures valid under weak scattering regimes both for small and large scatterers, namely Born and Rytov approximations. Although the problem is strongly simplified under this assumption, the study of linearized inverse scattering problems allows understanding, often by analytical findings, the role of fundamental parameters in the reconstruction capabilities of an imaging method, under adopted measurement configurations, such as the frequency, the number and configuration of probes, etc. Moreover, linearized approaches can be practically useful also when the working hypotheses underlying the weak scattering regime are not fully satisfied, that is when one wants to pursue only a qualitative characterization through microwave imaging.

The paper is structured as follows. In Section 2, the mathematical formulation of the scattering problem is given with respect to the scalar transverse magnetic (TM) 2D case. In Section 3, the linearized imaging procedure through OAM probing fields and Born and Rytov approximations are introduced. In Section 4, the proposed imaging strategy is validated against numerical examples. The conclusions end the paper.

## 2. Mathematical Formulation of the Inverse Scattering Problem

We consider the two-dimensional inverse scattering problem dealing with the TM polarization (scalar formulation) wherein nonmagnetic scatterers ($\mu_{s}=\mu_{0}$) are embedded into a homogeneous background medium. The location and the electromagnetic properties inside the domain are unknowns, and the vector $\underline{r}\left(x,y\right)$ denotes the position inside the investigation domain Ω. Time-harmonic dependence $e^{j\omega t}$, with angular frequency ω=2πf, is assumed dropped, and bold text notation for the electric fields is used hereafter.

**Figure 1:**
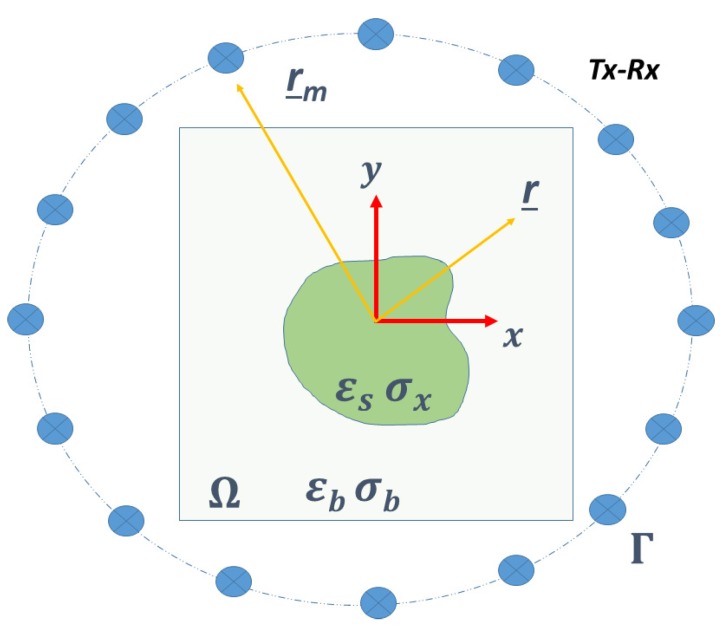
A sketch of the adopted multiview-multistatic measurement configuration to probe the region of interest by means of Tx-Rx primary sources (filamentary currents) placed on a circumference Γ of radius $r_{m}$.

To reconstruct the geometrical and dielectric properties of the scatterers, the investigated domain is probed with a set of incident fields $E_{i}\left({\underline{r}}_{v},\underline{r}\right)=E_{i}^{v}\left(\underline{r}\right)$, where ${\underline{r}}_{v}$ denotes the position of the primary sources (filamentary currents) placed outside the investigated area and *v* the indexing of the source. The interaction between the incident waves and the scatterers gives rise to a secondary field that is measured by receivers located at ${\underline{r}}_{m}\in \Gamma$, still outside the investigation domain. A sketch of the adopted measurement configuration is reported in Figure 1.

As is well known, the total field $E_{t}^{v}\left(\underline{r}\right)$ and the incident field $E_{i}^{v}\left(\underline{r}\right)$ must satisfy the following Helmholtz Equations:

(1) $$\begin{array}{c} \left[\nabla^{2}+k^{2}\left(\underline{r}\right)\right]E_{t}^{v}\left(\underline{r}\right)=0 \end{array}$$

(2) $$\begin{array}{c} \nabla^{2}+k_{b}^{2}]E_{i}^{v}\left(\underline{r}\right)=0 \end{array}$$

where $k(\underline{r})$ is the wavenumber in Ω and $k_{b}=\omega \sqrt{\varepsilon_{0}\varepsilon_{b}\mu_{0}}$ is the wavenumber of the homogeneous embedding background medium having complex permittivity $\varepsilon_{b}=\varepsilon_{b}^{{}^{\prime }}-j\frac{\sigma_{b}}{\omega \varepsilon_{0}}$. On the other hand, the scattered field, defined as $E_{s}^{v}\left(\underline{r}\right)=E_{t}^{v}\left(\underline{r}\right)-E_{i}^{v}\left(\underline{r}\right)$, satisfies the following Helmholtz Equation:

(3) $$\left[\nabla^{2}+k_{b}^{2}\left(\underline{r}\right)\right]E_{s}^{v}\left(\underline{r}\right)=-k_{b}^{2}\chi \left(\underline{r}\right)E_{t}^{v}\left(\underline{r}\right)$$

where the contrast function χ, which relates, at a given frequency ω, the properties of the unknown anomalies to those of the background medium, is defined as:

(4) $$\chi \left(\underline{r}\right)=\frac{\varepsilon_{s}\left(\underline{r}\right)}{\varepsilon_{b}}-1$$

with $\varepsilon_{s}=\varepsilon_{s}^{\prime }-j\frac{\sigma_{s}}{\omega \varepsilon_{0}}$ the complex dielectric permittivity of the scatterer. By means of the vector potential theory, the equations governing the scattering phenomenon can be conveniently expressed through a couple of integral Equations:

(5) $$\begin{array}{ccc} E_{s}^{v}\left({\underline{r}}_{m}\right) & = & k_{b}^{2}\;\int \int_{\Omega }g\left({\underline{r}}_{m},{\underline{r}}^{\prime }\right)\chi \left({\underline{r}}^{\prime }\right)E_{t}^{v}\left({\underline{r}}^{\prime }\right)d{\underline{r}}^{\prime }\;\;\;\;\;\;{\underline{r}}_{m}\in \Gamma ,\;\;\;v=1,\ldots ,V \end{array}$$

(6) $$\begin{array}{ccc} E_{t}^{v}\left(\underline{r}\right)-E_{i}^{v}\left(\underline{r}\right) & = & k_{b}^{2}\;\int \int_{\Omega }g\left(\underline{r},{\underline{r}}^{\prime }\right)\chi \left({\underline{r}}^{\prime }\right)E_{t}^{v}\left({\underline{r}}^{\prime }\right)d{\underline{r}}^{\prime }\;\;\;\;\;\;\underline{r}\in \Omega ,\;\;\;v=1,\ldots ,V \end{array}$$

where $g\left(\underline{r},{\underline{r}}^{\prime }\right)=-\frac{j}{4}H_{0}^{\left(2\right)}(k_{b}|\underline{r}-{\underline{r}}^{\prime }\left|\right)$ is the scalar Green function of the homogeneous background, in which ${\underline{r}}^{\prime }$ and $\underline{r}$ denote the generic source point in Ω and the observation point in Γ or Ω, respectively. Finally, $H_{0}^{\left(2\right)}\left(\cdot \right)$ is the Hankel function of zero order and second kind. The Green function is the kernel of the radiation operators $A_{e}\left[\cdot \right]:L^{2}\left(\Omega \right)\to L^{2}\left(\Gamma \right)$ and $A_{i}\left[\cdot \right]:L^{2}\left(\Omega \right)\to L^{2}\left(\Omega \right)$, which relate the induced contrast source $J^{v}\left(\underline{r}\right)$$=\chi (\underline{r})$$E_{t}^{v}\left(\underline{r}\right)$ to the field scattered in Γ and in Ω, respectively. Equations (5) and (6) are first and second kind Fredholm equations and are also known as data and state equations. According to the above, the inverse scattering problem is cast as the retrieval of the unknown contrast χ$\left(\underline{r}\in \Omega \right)$ from measured scattered field $E_{s}^{v}\left({\underline{r}}_{m}\in \Gamma \right)$ and known incident fields $E_{i}^{v}\left(\underline{r}\in \Omega \right)$.

## 3. Linear Imaging with OAM Incident Fields

As stated above, the solution of the problems Equations (5) and (6) entails facing a non-linear problem, since the total electric field has to be also retrieved for each transmitting antenna. To overcome this drawback, the first order linearized problem has been proposed in the past both for penetrable and impenetrable media. In particular, the Born and Rytov approximations allow tackling imaging of small and large weak scattering systems, provided that the deviation of the dielectric properties of anomalies, with respect to those of the background medium, keeps very small [24].

The linearized approach under Born approximation entails the solution of the following integral Equation:

(7) $$\begin{array}{ccc} E_{s}^{v}\left({\underline{r}}_{m}\right) & = & k_{b}^{2}\;\int \int_{\Omega }g\left({\underline{r}}_{m},{\underline{r}}^{\prime }\right)\chi \left({\underline{r}}^{\prime }\right)E_{inc}^{v}\left({\underline{r}}^{\prime }\right)d{\underline{r}}^{\prime }\;\;\;\;\;\;{\underline{r}}_{m}\in \Gamma ,\;\;\;v=1,\ldots ,V \end{array}$$

where the total field is substituted by the incident one, neglecting the effect of the scattering system on the total field. Similar considerations can be applied for the Rytov approximation, where the Equation to be solved turns out to be:

(8) $$\begin{array}{ccc} \Phi_{s}^{v}\left({\underline{r}}_{m}\right) & = & \frac{k_{b}^{2}}{E_{inc}^{v}\left({\underline{r}}_{m}\right)}\;\int \int_{\Omega }g\left({\underline{r}}_{m},{\underline{r}}^{\prime }\right)E_{inc}^{v}\left({\underline{r}}^{\prime }\right)\chi \left({\underline{r}}^{\prime }\right)d{\underline{r}}^{\prime }\;\;\;\;\;\;{\underline{r}}_{m}\in \Gamma ,\;\;\;v=1,\ldots ,V \end{array}$$

with $\Phi_{s}^{v}\left(r_{m}\right)$ the complex scattered phase [24], and $E_{inc}^{v}\left({\underline{r}}_{m}\right)$ the value of the incident fields at the measurement points. In both Equations (7) and (8), the incident field is commonly given by a sequential illumination of single filamentary currents placed in the near- or far-field of the imaging domain. The scattered field is collected by all the other antennas working as receivers when only one is acting as a transmitter. This is the standard scattering experiment procedure in a tomographic microwave imaging system. Hereafter, we refer to such a scheme of data gathering as “sequential illumination”.

On the other hand, we want to exploit OAM incident fields properly generated by a progressive phase change in circular array elements, that is:

(9) $$E_{inc}^{\ell }\left(\underline{r}\right)=-\frac{j}{4}\sum_{v=0}^{V-1}H_{0}^{\left(2\right)}(k_{b}|{\underline{r}}_{v}-\underline{r}\left|\right)e^{j\ell \varphi_{v}},\;\;\;\varphi_{v}=\frac{2v\pi }{V},\;\;\;\ell =0,\ldots ,\ell_{max}$$

**Figure 2:**
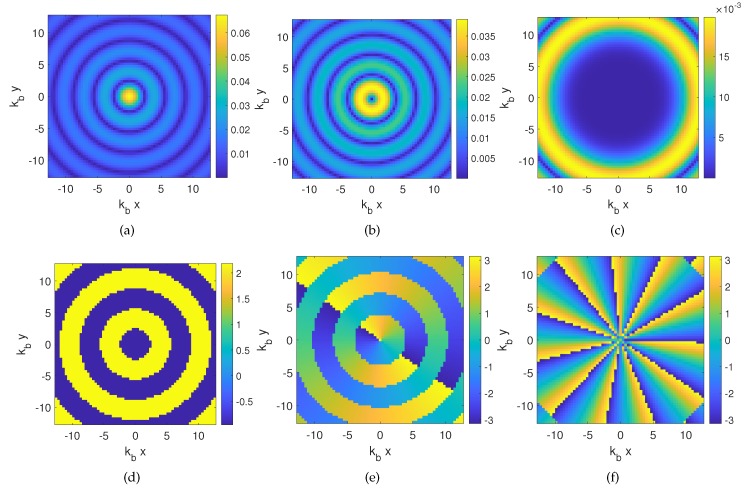
Amplitude (not normalized) and phase distribution of the OAM incident fields used to probe the imaging domain with a circular array of V = 36 filamentary currents. (**a**),(**d**) ℓ=0; (**b**),(**e**) ℓ=1; (**c**),(**f**) ℓ=11.

According to Equation (9), the investigation domain is illuminated through a set of different *ℓ*-order OAM fields rather than each single filamentary current placed at different angular positions. In a first approximation, without prior information on the scattering system, the maximum OAM order is related to the electrical dimension of the investigation domain, being |ℓ|≃βa, with $\beta =Re[k_{b}]$ and *a* the radius of the minimum convex hull enclosing the investigation domain. Incident fields arising from Equation (9) for three different orders are shown in Figure 2.

The data Equation we are going to consider hereafter is formally the same of Equations (7) and (8), with the corresponding scattered field collected under simultaneous illumination given by the incident fields Equation (9). In such a way, the role of the $v^{\mathrm{th}}$ illuminations is exchanged with the role of the $\ell^{\mathrm{th}}$ OAM order used to probe the investigation domain Ω. Therefore, as commonly done, the linearized tomography problem can still be solved in a regularized fashion. In this respect, we exploit the standard truncated singular value decomposition (TSVD) method [13], wherein the number $N_{T}$ of the relevant singular values to be used in the reconstruction formula is simply chosen by the cutoff of the singular values below the threshold of 20 dB lower than the maximum one.

## 4. Numerical Benchmarks

In order to investigate the opportunity to perform microwave imaging by means of OAM incident fields, we perform some proof-of-concept examples, under the weak scattering regime underlying both the Born and Rytov approximations.

The first example is concerned with a circular scatterer with radius $0.3\lambda_{b}$ and relative permittivity $\varepsilon_{s}=1.2-j0.06$, located at r=(0,0) in an investigation domain of $4\lambda_{b}\times 4\lambda_{b}$. The background medium is the vacuum, and V=2βa+1=37 antennas are used to illuminate the scenario [25] at a distance $r_{v}=40\lambda_{b}$. Accordingly, the same number *M* of measurement points, at the same distance ($r_{m}=r_{v}$), are used to probe the scattered field, as commonly done in any experimental microwave imaging apparatus. Using Equation (9), we generate OAM incident fields up to the order $|\pm \ell_{max}|=18$. Accordingly, the forward problem is solved by means of a method-of-moments (MoM) based solver by properly discretizing the investigation domain into $N_{c}=65\times 65$ cells, according to the Richmond rule [26]. Finally, the scattered field data are corrupted with an additive white Gaussian noise (AWGN) of SNR=20dB. The reconstruction results are evaluated by the standard metric based on the least squares mean error, which is defined as $err=\frac{\left|\right|\chi_{act}-\chi_{rec}{\left|\right|}^{2}}{\left|\right|\chi_{act}{\left|\right|}^{2}}$, wherein $\chi_{act}$ and $\chi_{rec}$ stem for the actual and the reconstructed contrast profile, respectively.

**Figure 3:**
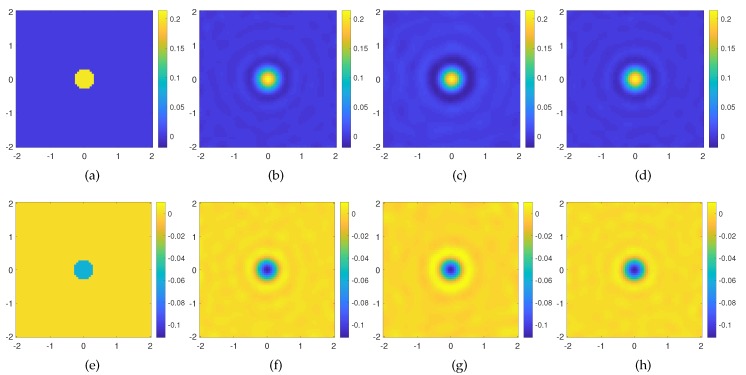
Reconstruction of a small weak circular scatterer through the Born approximation. (**a**) Real and (**e**) imaginary part of the actual contrast profile; (**b**) real and (**f**) imaginary part of the retrieved contrast profile for ℓ=0,±[1−18], err=0.2028 with a cutoff value in the TSVD equal to $N_{T}$ = 252; (**c**) real and (**g**) imaginary part of the retrieved contrast profile for ℓ=0,±[1−3], err=0.2384 with a cutoff value in the TSVD equal to $N_{T}$=142; (**d**) real and (**h**) imaginary part of the retrieved contrast profile using V=M=37 equispaced filamentary currents err=0.2020 with a cutoff value in the TSVD equal to $N_{T}$ = 252. The axes of the imaging domain are expressed in background wavelengths.

The reconstruction results using orders ℓ=0,±[1−18] are shown in Figure 3b–f. As can be seen, the method is able to retrieve the contrast correctly both in its real and imaginary part. After that, we consider only the first four lowest order (ℓ=0,±[1−3]) in solving the Born equation, and as shown in Figure 3c,g, the result is still good in terms of the reconstruction capability, since the reconstruction error is slightly larger than the previous one and mainly related to background reconstruction artifacts. On the other hand, it is worth noting that the dimension of the scattering matrix operator is $N_{c}\times \left(7\times M\right)$ for ℓ=[−3,+3] and $N_{c}\times \left(37\times M\right)$ for ℓ=[−18,+18], with $N_{c}={\left(65\right)}^{2}$ in both cases. If we take into account that also for the multiview-multistatic configuration, the matrix has dimension Nc×(V×M) (V=M=37) too, the computational advantage in the SVD numerical evaluation is not negligible in the case of few OAM modes. For the sake of completeness, the reconstruction performed with the standard multiview-multistatic field data acquisition is shown Figure 3d–h, and as expected, it is fully comparable with those achieved by means of the OAM incident fields.

**Figure 4:**
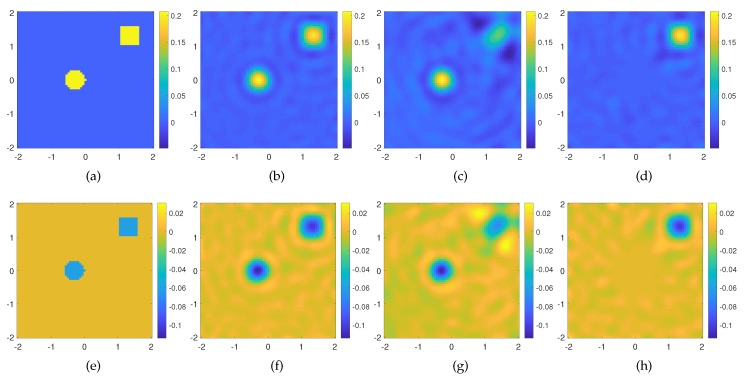
Reconstruction of a two small weak scatterers through the Born approximation. (**a**) Real and (**e**) imaginary part of the actual contrast profile; (**b**) real and (**f**) imaginary part of the recovered contrast profile for ℓ=0,±[1−18], err=0.2241 with a cutoff value in the TSVD equal to $N_{T}$ = 252; (**c**) real and (**g**) imaginary part of the recovered contrast profile for ℓ=0,±[1−4], err=0.4017 with a cutoff value in the TSVD equal to $N_{T}$ =166; (**d**) real and (**h**) imaginary part of the recovered contrast profile for ℓ=±[6−11] (without the lowest order modes), err=0.60 with a cutoff value in the TSVD equal to $N_{T}$ = 187. The axes of the imaging domain are expressed in background wavelengths.

In the second example, we consider a scattering system made of two off-centered targets embedded in the same investigation domain described for the previous example. The targets are shaped as a circle and a square scatterers, with permittivity $\varepsilon_{s}=1.2-j0.06$ and leading dimension $0.6\lambda_{b}$; see Figure 4a,e. We looks for the solution of the Born equation in three different cases. In the first case, we exploit all the OAM incident fields (ℓ=0,±[1−18]) needed to probe the entire domain, whereas in the second case, only the lowest order OAM incident fields (ℓ=0,±[1−4]), and finally, only the highest order ones (ℓ=±[6−11]). As can be seen in Figure 4, the reconstruction accounts for the whole scattering system in the first case (see Figure 4b,f), only the innermost scatterer in the second case (see Figure 4c,g), and only the square target in the third case (see Figure 4d,h). From these results, it is evident as the topological properties of the different OAM orders used to probe the domain are able to image targets whose support is mainly illuminated by the OAM rings (cores) of given order.

**Figure 5:**
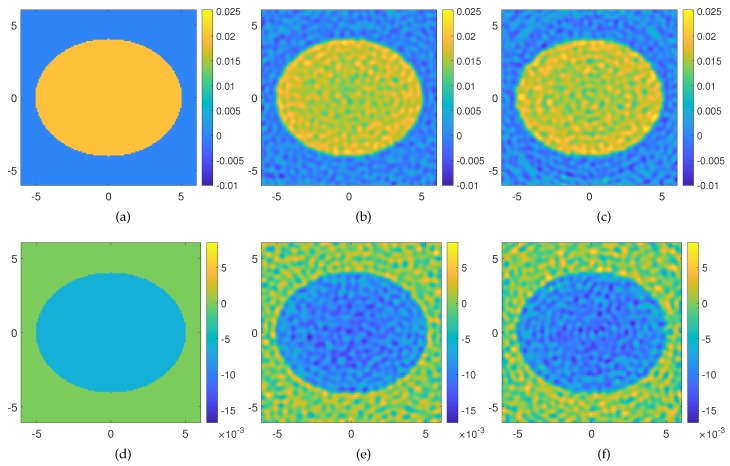
Reconstruction of a large circular scatterer through the Rytov approximation exploiting OAM fields generated in the far-field (${\underline{r}}_{m}=100$) m and near-field (${\underline{r}}_{m}=9$) m of the imaging domain. The OAM orders used are ℓ=0,±[1−35]. (**a**) Real and (**d**) imaginary part of the actual contrast profile; (**b**) real and (**e**) imaginary part of the retrieved contrast profile in the far-field measurement configuration, reconstruction err=0.1108 with a cutoff value in the TSVD reconstruction $N_{T}$ = 1856; (**c**) real and (**f**) imaginary part of the retrieved contrast profile in the near-field measurement configuration, reconstruction err=0.1267 with a cutoff value in the TSVD reconstruction $N_{T}$ = 1557. The axes of the imaging domain are expressed in background wavelengths.

Finally, the third example is concerned with a large lossless elliptically shaped target with semi-axes $5\lambda_{b}$ and $4\lambda_{b}$, respectively, embedded in an imaging domain of $12\lambda_{b}\times 12\lambda_{b}$ discretized into $N_{c}=129\times 129$ cells. The background is the vacuum, and the permittivity of the target is changed 2% with respect to the permittivity of the vacuum, while $Im\{\varepsilon_{s}\}=-0.006$. For such kinds of objects, the Rytov approximation holds true. According to the “rule of thumb” suggested by the electromagnetic field degrees of freedom [25], the domain needs to be probed by means of V=107 equispaced filamentary currents placed in the far-field of the imaging domain ($r_{m}=120\lambda_{b}$), and the corresponding scattered fields are collected through M=107 measurement points. We consider the solution of the forward problem with a set of OAM incident fields up to $|\pm \ell_{max}|=30$ and solve the underlying inverse problem by means of the Rytov Equation (8). In this case also, the scattered field is corrupted with AWGN of SNR = 20 dB. The reconstruction results are shown in Figure 5b–e and allow appraising the dielectric features of the target. It is worth noting that, if the SVD of the multiview-multistatic scattering matrix, of dimensions $\left(N_{c}\right)\times \left(M\times V\right)$, is computed via MATLAB on a standard CPU Intel Core i7 8GB RAM, it results in an “out-of-memory” warning. Instead, by using the proposed OAM based inversion, a meaningful result is found without the need for higher performance computers. Furthermore, for this example, we consider also reconstruction by processing the scattered field gathered in the near-field (though non-reactive zone) of the imaging domain by setting the distance of the Tx-Rx probes at $r_{m}=9\lambda$, namely the minimum circle enclosing the surveyed area. As we can appraise from Figure 5c–f, the reconstruction results are fully comparable as the reconstruction errors attain the same values in both considered cases.

## 5. Conclusions

The use of OAM incident fields in linearized diffraction tomography has been investigated and analyzed in this paper. The main conclusions are concerned with the possibility to adopt alternative measurement setup that give additional flexibility in performing tomographic imaging.

As an example, this is the case when the investigated domain contains undesired scatterers that should be neglected in the reconstruction process, such as, for example, in those approaches wherein part of the electromagnetic features of the scene is known, such as non-destructive testing (NDT) for the detection of faults in an “undesired” background. Indeed, this can be done via hardware, without resorting to computationally heavy differential (or distorted) imaging procedures, wherein the knowledge of the Green function is also required. Another possible context of interest is in subsurface imaging where the goal may be to address the inversion strategy at a given depth and/or location, on the base of a priori available information.

On the other hand, in all those applications concerned with the detection of small scatterers, OAM probing fields can allow to significantly reduce gathering time and computational burden, as only few modes have to be exploited to achieve satisfactory reconstructions. Indeed, the size of the multiorder-multistatic scattering operator is often smaller than the multiview-mutistatic counterpart, depending on the dimension of the scattering system, and hence on the $|\ell_{max}|$ order used to probe the scenario. This may be of particular interest for the development of fast 3D tomographic approaches with reduced computational burden.

As main drawback, OAMs imply a complication of the feeding network (phase shifters and possibly amplifiers) that can be traded, on the other hand, with a lower acquisition time through digital beamforming networks that avoid sequentially transmitting antennas and switching circuitry. Last, but not least, it is worth noticing that, when no a priori information is available about the scattering system, it is even possible to scan the OAM cores along some directions over the investigation domain, according to the well known phased array theory.
